# Proxy-based modeling of indoor air pollution exposure and nonlinear health patterns in biomass-dependent households using machine learning

**DOI:** 10.3389/fpubh.2026.1863424

**Published:** 2026-07-01

**Authors:** Adeyinka Odebode, Olusiji Lasekan, Ayorinde Ogundele, Héctor H. Silva, Boluwatife Adigun

**Affiliations:** 1 Bishop Barham University College, Uganda Christian University, Kabale, Uganda; 2 Universidad Católica de Temuco, Temuco, Chile; 3 Universidad de La Frontera, Temuco, Chile; 4 ART Clinic, Regional Referral Hospital, Kabale, Uganda

**Keywords:** biomass fuels, exposure modeling, household air pollution, machine learning, nonlinear health effects

## Abstract

This study seeks to address critical gaps in household air pollution (HAP) exposure assessment in low- and middle-income settings by applying a proxy-based exposure reconstruction framework supported by machine learning to develop an exploratory proxy-based exposure index and examine associations with nonlinear health patterns in Kabale, Uganda. A quantitative cross-sectional survey (*N* = 275) was conducted to capture behavioral, environmental, and socio-demographic proxies of exposure, including fuel type, cooking duration, ventilation, and cooking location. A latent exposure index was developed using Principal Component Analysis and validated with Random Forest and Gradient Boosting models, while nonlinear exposure–health relationships were analyzed using ML classifiers and household typologies identified through *K*-Means clustering. The findings reveal that exposure is driven primarily by behavioral factors, particularly cooking duration, rather than fuel type alone. Nonlinear modeling suggested possible threshold-like and plateauing patterns in self-reported health outcomes, with acute symptoms such as eye irritation appearing at lower proxy-based exposure levels and chronic respiratory distress showing possible plateauing at higher exposure levels. Clustering analysis identifies a large high-risk group characterized by prolonged indoor biomass use and elevated symptom burden. ML models outperform traditional regression approaches in predicting chronic symptoms but show limited performance for clinical diagnoses. These findings suggest that proxy-based HAP exposure patterns may be multidimensional and potentially nonlinear and demonstrate the value of ML in proxy-based environments, emphasizing that policy interventions must move beyond fuel switching to address behavioral practices and structural constraints shaping exposure.

## 1 Introduction

Household air pollution (HAP) remains a major environmental health challenge in low- and middle-income countries, particularly in regions where households rely on biomass fuels such as charcoal, firewood, dung, and crop residues for cooking and heating. Combustion of these fuels releases substantial concentrations of fine particulate matter (PM₂.₅), carbon monoxide, and other harmful pollutants that contribute to respiratory and cardiovascular morbidity, especially in poorly ventilated indoor environments (1–3). In sub-Saharan Africa, dependence on biomass fuels remains widespread due to limited access to clean energy alternatives, resulting in continued exposure to household-generated pollutants and a disproportionate burden of respiratory disease and premature mortality (4–6). Despite ongoing efforts to promote cleaner cooking technologies, household air pollution continues to represent a significant public health concern across resource-constrained settings (7, 8).

A major challenge in HAP research is the limited availability of direct indoor air quality monitoring in low-resource settings. Measurement of pollutants such as PM₂.₅, carbon monoxide, and nitrogen oxides often requires specialized equipment, technical expertise, and sustained financial investment, which are frequently unavailable in many communities (9–11). Consequently, researchers commonly rely on household-level proxy indicators—including fuel type, cooking duration, ventilation conditions, smoke intensity, soot presence, and cooking location—to estimate exposure burden. While these indicators provide valuable information, they are often analyzed independently, potentially overlooking the cumulative, interacting, and nonlinear nature of real-world exposure environments (12, 13). Such approaches may inadequately represent the multidimensional processes through which household behaviors, environmental conditions, and structural characteristics jointly influence exposure and health outcomes.

The present study is grounded in three complementary theoretical perspectives that support a multidimensional understanding of household air pollution exposure. The Environmental Health Risk Framework conceptualizes the pathway linking pollution sources, exposure pathways, dose accumulation, and adverse health outcomes, providing a foundation for understanding how biomass combustion contributes to respiratory and cardiovascular disease (3, 14, 15). Cumulative Exposure Theory further emphasizes that chronic health effects emerge from repeated and prolonged exposure rather than isolated pollution events, highlighting the importance of cumulative exposure assessment in environmental health research (6, 16, 17). In addition, Complex Systems Theory suggests that exposure and health outcomes emerge from interactions among multiple behavioral, environmental, and socioeconomic determinants rather than from single risk factors operating independently (13, 18). Together, these perspectives support the use of composite exposure indicators and nonlinear analytical approaches capable of capturing the complexity of household pollution environments.

**Figure 1:**
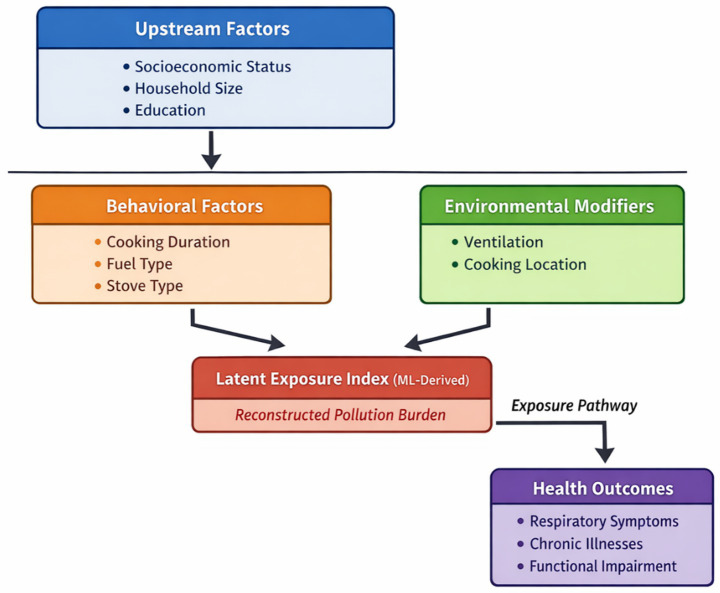
Conceptual framework illustrating the pathways linking upstream socio-demographic factors, behavioral practices, and environmental conditions to latent household air pollution exposure and health outcomes.

Based on these theoretical foundations, this study conceptualizes household air pollution exposure as a latent construct shaped by upstream socio-demographic factors, behavioral practices, and environmental conditions. Socioeconomic status, household size, and educational attainment influence household decision-making and resource access, which in turn affect behaviors such as fuel selection, stove use, and cooking duration. Environmental conditions, including ventilation and cooking location, further modify pollutant accumulation and dispersion within households. Rather than treating these determinants as isolated variables, the proposed framework assumes that they interact to generate an underlying exposure burden that subsequently influences respiratory symptoms, clinical health conditions, and functional impairment. This systems-based perspective provides the conceptual basis for reconstructing latent exposure through proxy-based machine learning methods and examining its relationship with health outcomes (see Figure 1).

Previous research has demonstrated that household air pollution exposure is influenced by a complex combination of behavioral, environmental, and structural factors. While biomass fuels generate substantial emissions, actual exposure depends on factors such as cooking duration, cooking location, housing structure, and ventilation effectiveness (19–21). Longer cooking durations increase cumulative exposure, whereas indoor cooking and inadequate ventilation may trap pollutants and facilitate their diffusion into living spaces. Furthermore, perceived ventilation conditions do not always correspond to actual pollutant removal efficiency, highlighting the complexity of exposure assessment within household environments (20, 22).

To address these challenges, environmental health research has increasingly adopted proxy-based and data-driven approaches for exposure assessment. Proxy-based models estimate exposure using indirect indicators such as fuel-use patterns, proximity to pollution sources, or household characteristics and are particularly valuable in settings where direct pollutant monitoring is unavailable or impractical (23, 24). Data-driven approaches, including generalized additive models, Random Forests, support vector machines, neural networks, and spatiotemporal models, leverage statistical and machine learning techniques to identify complex exposure patterns and improve prediction accuracy (25, 26). These approaches have become increasingly important in epidemiological research, environmental justice studies, and public health policy because they facilitate the identification of vulnerable populations and support targeted interventions (23, 27, 28). Nevertheless, challenges remain regarding proxy validity, missing data, transferability across settings, and the representation of cumulative exposure processes (29).

Despite growing recognition that household air pollution exposure is shaped by multiple interacting determinants, important methodological gaps remain. Many household air pollution studies continue to rely on single exposure indicators or conventional linear analytical approaches that may inadequately represent cumulative exposure burden and nonlinear exposure–health relationships. Similarly, most machine learning applications in air pollution research focus on ambient pollution forecasting, sensor calibration, or spatiotemporal prediction rather than household-level proxy-based exposure reconstruction. Consequently, limited research has integrated multiple household exposure proxies into a composite latent exposure index, examined potential nonlinear exposure–health patterns, and simultaneously identified household exposure typologies within a unified analytical framework. This limitation is particularly important in low-resource settings where direct pollutant monitoring is unavailable and proxy-based methods represent the primary means of exposure assessment.

To address these gaps, this study develops an exploratory proxy-based exposure modeling framework supported by machine learning to investigate household air pollution exposure among biomass-dependent households in Kabale, Uganda. Specifically, the study aims to: (1) construct a composite latent exposure index from household-level proxy indicators; (2) examine potential nonlinear associations between proxy-based exposure and self-reported health outcomes; and (3) identify household exposure typologies that may inform targeted public health intervention strategies in resource-constrained settings.

## 2 Methods

### 2.1 Study design and setting

This study employed a quantitative cross-sectional household survey design to assess indoor air pollution exposure and associated health outcomes among households in Kabale District, South-Western Uganda. Kabale is characterized by a mixed rural–urban setting, with the majority of households relying on biomass fuels, particularly charcoal, for daily cooking and heating needs. The survey targeted households using charcoal stoves, reflecting the widespread dependence on this fuel source within the study area. Data were collected at a single point in time using structured questionnaires administered to household members, capturing quantifiable information on socio-demographic characteristics, cooking practices, environmental conditions, and self-reported health outcomes.

The study setting is marked by prevalent indoor cooking practices, often conducted in enclosed or semi-enclosed spaces with varying levels of ventilation, which significantly influences exposure to household air pollutants. Charcoal use dominates due to its relative affordability and accessibility compared to cleaner energy alternatives, contributing to sustained indoor emission of particulate matter and other harmful pollutants. This combination of high charcoal dependence and indoor cooking behaviors makes Kabale an appropriate context for investigating complex exposure patterns and their health implications in resource-constrained environments.

### 2.2 Sampling and data collection

Households were selected from communities within Kabale District using a non-probability household sampling approach based on accessibility, household availability, and willingness to participate. The sampling strategy was therefore exploratory and should not be interpreted as statistically representative of all households in Kabale District. Eligible households were those using household cooking systems, particularly biomass or charcoal-based cooking, and with an adult household member available to provide information on cooking practices, household characteristics, and health symptoms. The questionnaire was completed by an adult respondent who was familiar with household cooking routines and indoor environmental conditions, preferably the primary cook or another household member directly involved in cooking activities. Data were collected at a single point in time using a structured questionnaire administered to household respondents. Interviewers were oriented on the study objectives, questionnaire items, informed consent procedures, confidentiality, and standardized administration of questions prior to data collection. This training was intended to minimize interviewer variability, improve consistency in data collection, and reduce measurement bias. Because the study relied on cross-sectional and self-reported household data, the findings should be interpreted as exploratory and context-specific rather than fully generalizable to all households in the district.

### 2.3 Data sources and variables

The primary data source for this study was a quantitative cross-sectional household survey conducted in Kabale, Uganda (*N* = 275 valid responses), designed to capture a multidimensional profile of household air pollution (HAP) exposure, associated health outcomes, and key socio-demographic characteristics. Exposure was operationalized using a composite set of behavioral and environmental proxies rather than single indicators, including primary cooking fuel, daily cooking duration, specific cooking hours, cooking location (indoor, outdoor, or separate kitchen), ventilation adequacy, soot presence, and perceived smoke intensity during cooking, consistent with established approaches to proxy-based exposure assessment in low-resource settings (30–32). Health outcomes were assessed across a gradient of severity, incorporating self-reported respiratory symptoms such as coughing, wheezing, and shortness of breath, alongside ordinal measures of breathing difficulty frequency and self-reported clinical diagnoses of respiratory and cardiovascular conditions, following standard survey-based health assessment frameworks in environmental health research (33, 34). To account for potential confounding, the analysis further incorporated key socio-demographic covariates—including age, sex, educational attainment, and household income—recognizing their established influence on both exposure patterns and baseline health vulnerability (35, 36).

### 2.4 Data preprocessing

Rigorous data preprocessing was conducted to ensure the integrity of the machine learning pipeline and to minimize risks of data leakage and algorithmic bias. Categorical variables were carefully encoded based on their structure: ordinal variables (e.g., cooking duration, smoke intensity, and symptom frequency) were transformed into monotonic integer scales to preserve their inherent ranking, while nominal variables (e.g., cooking fuel and cooking location) were encoded using either one-hot or target encoding depending on algorithm requirements. Missing data were addressed using a median imputation strategy to reduce the influence of outliers and maintain distributional stability, with four records containing entirely missing values removed prior to analysis. The household income variable, which exhibited a high non-response rate (>90%), was retained selectively as an indicator variable, while greater emphasis was placed on more reliable socio-economic proxies such as education and occupation. Finally, to ensure balanced feature contribution—particularly for distance-based methods such as *K*-Means and variance-based techniques like Principal Component Analysis (PCA)—all continuous and ordinal exposure variables were standardized using *Z*-score normalization (mean = 0, standard deviation = 1) prior to model implementation.

### 2.5 Machine learning framework

The analytical framework employed a suite of machine learning (ML) techniques to construct exposure metrics, model nonlinear health relationships, and identify household typologies. To ensure methodological transparency, the analytical methods were selected based on the structure of the dataset, study objectives, sample size, interpretability requirements, and the exploratory nature of proxy-based exposure reconstruction. Because the study relied on cross-sectional household survey data rather than high-frequency sensor measurements, priority was given to methods that are robust to mixed variable types, capable of handling nonlinear relationships, interpretable for public health decision-making, and appropriate for moderate sample sizes. Table 1 summarizes the purpose, advantages, limitations, and justification for each method used.

**Table 1:** Summary of analytical methods and justification for selection.

Method	Purpose in this study	Advantages	Limitations	Justification for selection
Principal component analysis (PCA)	Construct baseline latent exposure score from correlated household proxy variables	Reduces dimensionality; creates composite exposure index; useful when direct pollutant measurements are unavailable	Assumes linear combinations of variables; sensitive to scaling; may not capture nonlinear latent structure	Selected because the exposure variables were correlated proxies and the aim was to create an interpretable composite index rather than estimate direct pollutant concentrations
Random Forest	Validate variable contributions and model nonlinear exposure–health relationships	Captures nonlinear interactions; robust to mixed predictors; provides feature importance; less sensitive to outliers	Less interpretable than regression; may overfit if not cross-validated	Selected because HAP exposure is multidimensional and likely nonlinear; also suitable for moderate-sized survey data
Gradient boosting	Model nonlinear exposure–health relationships and detect threshold/plateauing pattern	Strong predictive performance; captures complex nonlinear patterns; useful for partial dependence analysis	Sensitive to tuning; can overfit small datasets	Selected to complement Random Forest and test whether boosted ensemble learning better captured nonlinear health responses
*K*-Means clustering	Identify household exposure typologies	Simple, interpretable, scalable; useful for identifying exposure profiles	Requires pre-specified number of clusters; assumes spherical clusters; sensitive to scaling	Selected because the study aimed to classify households into practical exposure-risk groups for intervention targeting
SHAP	Explain model predictions and identify important exposure drivers	Improves interpretability; provides global and local feature attribution	Computationally intensive; explanations depend on fitted model quality	Selected to make ML outputs transparent and policy-relevant by identifying actionable drivers such as cooking duration and smoke intensity
Logistic/ridge regression	Traditional comparison models	Familiar, interpretable, useful baseline models	Limited ability to capture nonlinear and interaction effects	Included as benchmark models to assess whether ML improved prediction over conventional statistical approaches

Alternative approaches such as XGBoost, Support Vector Machines, generalized additive models, latent class analysis, Bayesian models, and structural equation modeling were considered; however, they were not prioritized because of the study’s moderate sample size, exploratory proxy-based design, need for interpretability, and emphasis on practical exposure classification rather than causal pathway estimation or high-dimensional prediction.

#### 2.5.1 Latent exposure construction

A composite “Latent Exposure Score” was constructed to quantify the cumulative HAP burden. Initial dimensionality reduction was performed using Principal Component Analysis (PCA) on the scaled exposure proxies, extracting the first principal component as the baseline continuous score. To validate the variable contributions to this score, Random Forest and Gradient Boosting regressors were trained to predict the latent score from the raw proxies, establishing the relative importance of temporal variables (cooking duration) versus material variables (fuel type). The resulting score should be interpreted as an exploratory proxy-based exposure index rather than a direct measurement of pollutant concentration, since no PM₂.₅, CO, or NO₂ monitoring data were available.

#### 2.5.2 Nonlinear health modeling

To explore potential nonlinear associations between the latent exposure score and self-reported health outcomes, ML classifiers, including Random Forest and Gradient Boosting, were applied to predict symptom occurrence and disease presence. These tree-based ensemble methods were selected because they can capture complex and potentially nonlinear patterns, including threshold-like or plateauing trends. In this study, a threshold-like effect refers to a point in the proxy-based exposure score where the predicted probability or severity of a health outcome begins to increase more noticeably, while a plateauing/saturation effect refers to a pattern where the predicted health response levels off despite further increases in the exposure score. Their predictive performance was compared with traditional Logistic Regression for binary outcomes and Ridge Regression for ordinal outcomes. Because the partial dependence plots were exploratory and did not include uncertainty intervals, nonlinear patterns were interpreted cautiously as suggestive associations rather than confirmed exposure–response thresholds.

#### 2.5.3 Clustering analysis

Unsupervised *K*-Means clustering was applied to the scaled exposure feature space to identify naturally occurring household typologies. To determine the optimal number of clusters, *K*-Means models were evaluated across multiple values of *k* (2–7) using silhouette scores and visual inspection of cluster separation. Although the highest silhouette score was observed at *k* = 5, the numerical differences between candidate solutions were small. Therefore, cluster selection was not based on silhouette score alone. The final *k* = 3 solution was retained because it offered the best balance between statistical adequacy, visual separation in PCA space, and practical interpretability. The three-cluster structure produced conceptually meaningful and clinically relevant household typologies—low exposure, moderate exposure, and high-risk indoor cookers—which aligned with the study objective of identifying actionable household exposure profiles for public health intervention. Solutions with larger *k* values produced smaller subgroups with limited interpretability and overlapping exposure characteristics, reducing practical usefulness for policy translation.

#### 2.5.4 Explainability

To ensure the ML models remained transparent and clinically interpretable, SHAP (SHapley Additive exPlanations) values were utilized. SHAP values provided localized feature attribution, answering two critical questions: (1) Which specific behavioral or environmental variables drive the composite exposure score? and (2) Which factors are the strongest predictors of disease and symptom onset? This allowed for the identification of critical intervention targets, such as cooking duration and indoor location.

### 2.6 Model validation

Robust validation protocols were implemented to minimize overfitting and ensure the generalizability of the machine learning models. All predictive models were evaluated using 5-fold stratified cross-validation, which preserves class distribution across folds and is particularly important for imbalanced outcomes such as clinical diagnoses. In addition, a stratified 75/25 train-test split was applied to assess model performance on unseen data, specifically for the generation of Receiver Operating Characteristic (ROC) curves. Model performance was evaluated using a combination of complementary metrics: Area Under the ROC Curve (AUC) served as the primary metric for binary classification tasks due to its robustness to class imbalance, accuracy was reported alongside AUC but interpreted cautiously in the context of rare outcomes, and Root Mean Squared Error (RMSE) was used to assess the precision of regression models predicting ordinal symptom severity (e.g., frequency of breathing difficulty and eye irritation).

To address class imbalance, classification performance was evaluated using multiple complementary metrics rather than accuracy alone. These included AUC, precision–recall AUC (PR-AUC), sensitivity/recall, specificity, precision, F1-score, and confusion matrices. For imbalanced outcomes such as clinical diagnosis, PR-AUC, sensitivity, specificity, and F1-score were prioritized over accuracy because accuracy may be inflated when the minority class is rare. Confidence intervals for AUC and PR-AUC were estimated using bootstrap resampling of the test set.

### 2.7 Model implementation and Hyperparameter settings

All analyses were implemented in Python using pandas, numpy, scikit-learn, matplotlib, and SHAP libraries. Prior to model fitting, categorical variables were encoded according to their measurement level, and continuous and ordinal exposure variables were standardized using *Z*-score normalization. A fixed random seed of 42 was used across all stochastic procedures to ensure reproducibility. Model development used a stratified 75/25 train-test split for holdout evaluation and 5-fold stratified cross-validation for classification tasks. Because the study was exploratory and based on a moderate sample size, hyperparameter tuning was limited; default scikit-learn settings were retained unless otherwise stated, and model performance was interpreted cautiously to avoid overfitting.

The implementation settings and key hyperparameters used for the Random Forest, Gradient Boosting, and *K*-Means models are reported explicitly in Table 2. These settings were selected to ensure analytical consistency, reproducibility, and comparability across models while minimizing overfitting in this exploratory analysis.

**Table 2:** Model implementation details and hyperparameter settings.

Method	Main settings reported	Implementation details
Random Forest Regressor	n_estimators = 150; criterion = squared_error; max_depth = None; min_samples_split = 2; min_samples_leaf = 1; random_state = 42	Used to estimate feature importance for the latent exposure index
Random Forest Classifier	n_estimators = 100; criterion = gini; max_depth = None; min_samples_split = 2; min_samples_leaf = 1; random_state = 42	Used for binary health outcome prediction and comparison with traditional models
Gradient boosting regressor	n_estimators = 100; learning_rate = 0.1; max_depth = 3; loss = squared_error; random_state = 42	Used for nonlinear exposure–health modeling and partial dependence analysis
Gradient boosting classifier	n_estimators = 100; learning_rate = 0.1; max_depth = 3; loss = log_loss; random_state = 42	Used for binary health outcome prediction and nonlinear classification
*K*-Means clustering	n_clusters = 3; init = k-means++; n_init = 10; max_iter = 300; tol = 1e-4; random_state = 42	Used to classify households into low, moderate, and high-risk exposure typologies

Based on silhouette performance and interpretability of the exposure profiles, the three-cluster solution was retained for subsequent household typology analysis. The silhouette scores as follows (see Table 3):

**Table 3:** Silhouette scores for *K*-Means clustering solutions.

Number of clusters (k)	Silhouette score
2	0.1366
3	0.1321
4	0.1347
5	0.1503
6	0.1446
7	0.1351

### 2.8 Ethical considerations

This study involved anonymous, minimal-risk household survey data and did not include clinical procedures, biological sampling, or collection of personally identifiable information. Formal institutional ethical review was not sought because the study was conducted as a low-risk observational household survey. Nevertheless, the research adhered to ethical principles for human subjects research. Written informed consent was obtained from all respondents prior to participation, participation was voluntary, and all responses were anonymized prior to analysis through removal of identifying information and assignment of randomized household identifiers.

## 3 Results

### 3.1 Descriptive statistics

This descriptive analysis of the Kabale household cohort (*N* = 275) highlights a critical disconnect between socio-demographic status and environmental health risk in Table 4.

**Table 4:** Population profile and exposure behaviors (*N* = 275).

Category and variable	*n* (%)
Population profile	
*Age group*	
15–30 years	213 (77.5%)
31–50 years	59 (21.5%)
>50 years/not stated	3 (1.1%)
*Sex*	
Male	139 (50.5%)
Female	128 (46.5%)
Other/not stated	8 (2.9%)
*Marital status*	
Single	188 (68.4%)
Married	78 (28.4%)
Other (divorced/widowed)	9 (3.3%)
*Education level*	
Tertiary	175 (63.6%)
Secondary and below	100 (36.4%)
*Primary occupation*	
Student	130 (47.3%)
Employed/business	135 (49.1%)
Peasant/farmer	10 (3.6%)
*Household size* (Mean ± SD)	3.5 ± 2.6
Exposure behaviors	
*Primary cooking fuel*	
Solid biomass (charcoal/firewood)	207 (75.3%)
Clean fuel (gas/electricity)	63 (22.9%)
Other	5 (1.8%)
*Daily cooking window*	
≤5 h	154 (56.0%)
>5 h	120 (43.6%)
*Cooking hours per day*	
≤3 h	193 (70.2%)
>3 h	82 (29.8%)
*Cooking location*	
Dedicated kitchen/outdoor	188 (68.4%)
Inside (living area)	87 (31.6%)
*Ventilation and emissions*	
Perceived as well ventilated	192 (69.8%)
Observed soot presence	191 (69.5%)
Mod/high smoke intensity	150 (54.5%)
*Improved cooking method*	
Uses improved method	240 (87.3%)
Does not use/not sure	35 (12.7%)

The descriptive statistics reveal a striking and policy-relevant paradox in household air pollution exposure within the study population. Contrary to conventional assumptions that HAP is primarily a problem of low education and rural poverty, the sample is predominantly young (77.5% under 30), highly educated (63.6% with tertiary education), and largely composed of students or employed individuals (96.4%), yet a substantial majority (75.3%) still rely on biomass fuels such as charcoal and firewood for cooking. This indicates that fuel choice is not driven by lack of awareness but by structural economic and access constraints, as cleaner fuels like gas and electricity remain underutilized (22.9%). Exposure is further intensified by behavioral patterns, where although many households report relatively short daily cooking durations (≤3 h), a large proportion operate within extended cooking windows exceeding 5 h, leading to prolonged low-intensity combustion and sustained pollutant release. This risk is compounded by spatial practices, with 31.6% of households cooking within living spaces rather than in separate kitchens or outdoors, facilitating direct indoor accumulation of pollutants. Critically, there exists a clear illusion of ventilation, as nearly 70% of respondents perceive their cooking environments as well ventilated, yet objective indicators contradict this perception: 69.5% report visible soot deposits and 54.5% experience moderate to high smoke intensity. Even among the 87.3% who report using “improved” cooking methods, persistent soot and smoke suggest these technologies are either inefficient or inadequately implemented. Overall, the findings depict a population that is educated but structurally constrained, engaging in high-risk energy behaviors that sustain indoor pollution exposure; thus, effective interventions must move beyond awareness campaigns and instead prioritize affordability and accessibility of clean fuels, alongside genuinely effective ventilation and stove technologies capable of reducing indoor particulate accumulation.

These characteristics indicate that the sample represents a specific “educated but exposed” subgroup rather than the full spectrum of biomass-dependent households in sub-Saharan Africa. Therefore, the findings should not be interpreted as representative of poorer, less educated, or more rural households, where exposure may be shaped by different economic constraints, cooking environments, fuel access, and health vulnerabilities.

### 3.2 Latent exposure index construction

**Figure 2:**
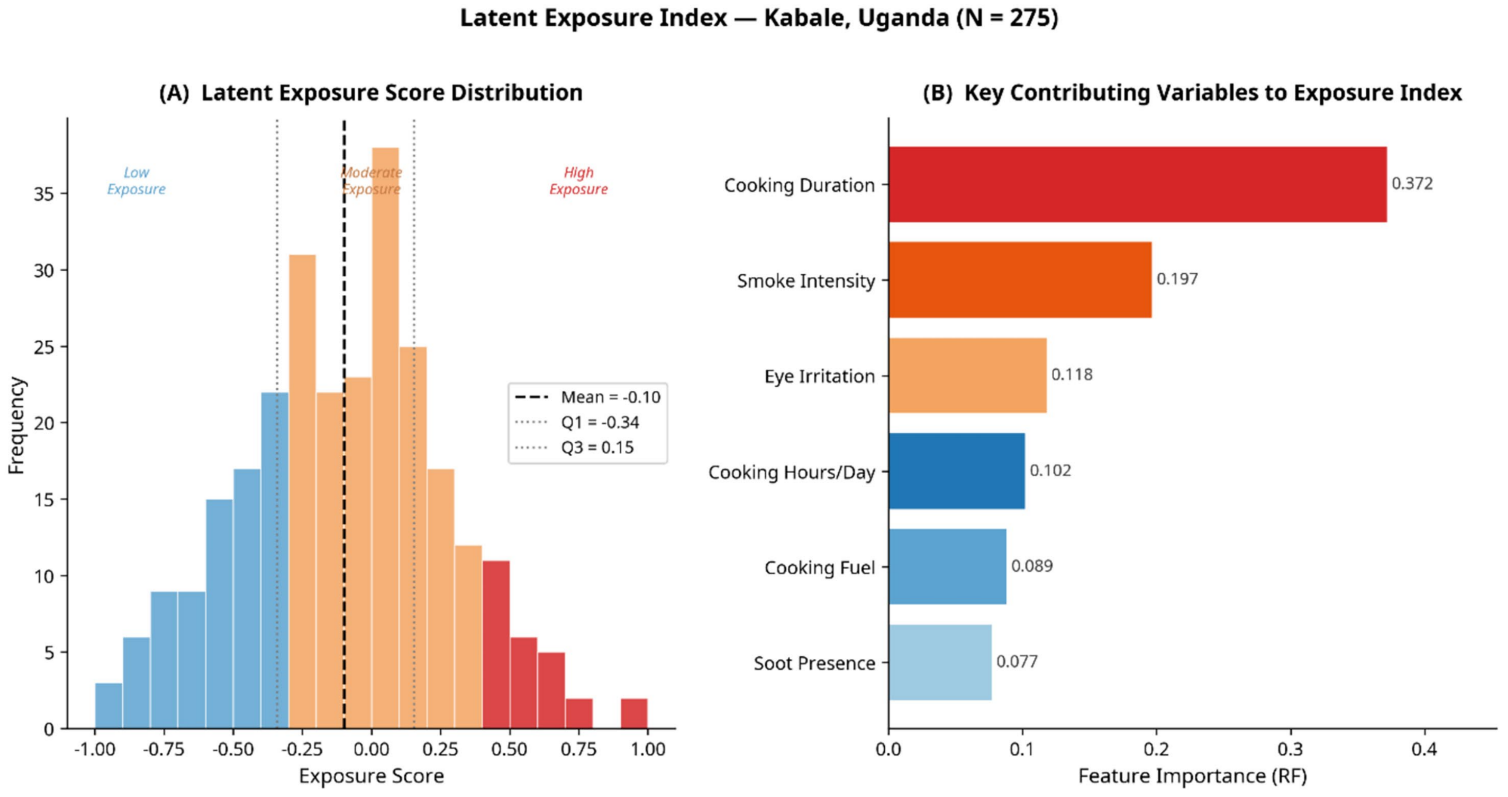
Latent Exposure Index Construction. **(A)** Distribution of latent exposure scores across households in Kabale, Uganda. **(B)** Key contributing variables to the proxy-based latent exposure index based on Random Forest feature importance.

Figure 2 shows the Latent Exposure Index for household air pollution in Kabale, Uganda (*N* = 275). The index was derived by integrating nine behavioral and environmental proxy variables into a composite score, normalized to a range of [−1, 1]. A Random Forest Regressor was then applied to extract the feature importance, revealing which variables most strongly drive the overall proxy-based exposure burden.

The latent exposure index results (Panel A and Panel B) reveal a clear and policy-relevant pattern in household air pollution dynamics. The distribution of exposure scores is approximately normal with a slight right skew, indicating that while most households cluster around moderate exposure levels (mean ≈ − 0.10), a distinct high-exposure tail exists, representing about 10.5% of households experiencing severe and compounded pollution risks. The majority (63.3%) fall within moderate exposure, while 26.2% exhibit relatively low exposure, reflecting protective behaviors such as shorter cooking durations or cleaner fuel use. This stratification highlights the presence of cumulative exposure gradients, supporting the idea that a subset of households is subjected to chronic, high-intensity pollution that would be obscured by single-variable assessments. Complementing this, the feature importance analysis (Panel B) demonstrates that temporal and experiential factors dominate exposure prediction, with cooking duration emerging as the most influential variable (0.372), followed by smoke intensity (0.197) and eye irritation (0.118), while traditional determinants such as cooking fuel (0.089) and soot presence (0.077) play comparatively smaller roles. These findings fundamentally challenge the conventional assumption that fuel type alone is associated with exposure, instead showing that how long pollutants are generated and how intensely they are experienced are more critical determinants of overall proxy-based exposure burden. Overall, the results confirm that household air pollution is a multidimensional and nonlinear phenomenon, and that machine learning–based composite indices are essential for capturing these dynamics. The implication is clear: interventions that focus solely on fuel switching or improved stoves may be insufficient unless they also address behavioral patterns—particularly prolonged cooking durations—that sustain high cumulative exposure in resource-constrained settings like Kabale.

### 3.3 Nonlinear exposure–health relationships

**Figure 3:**
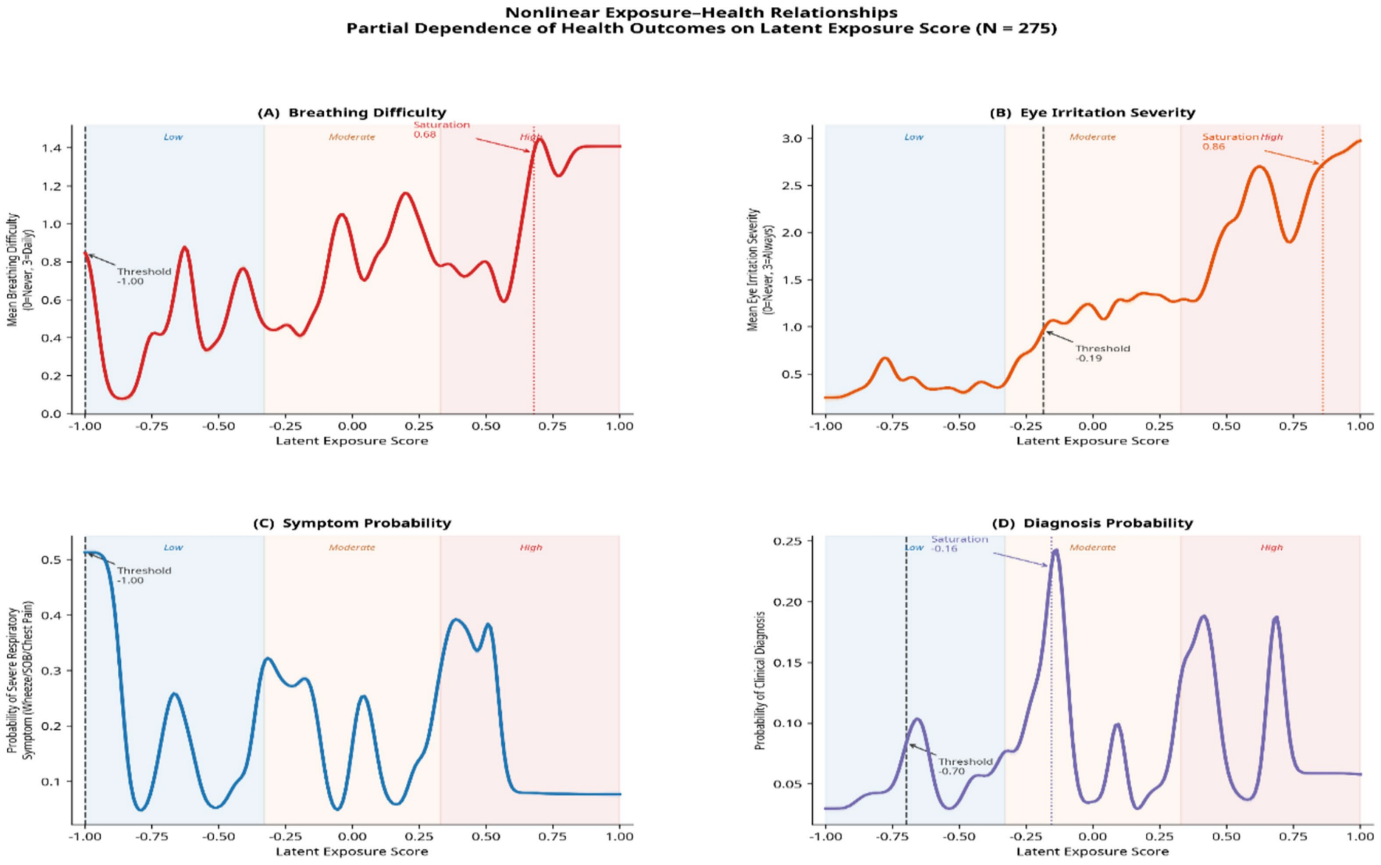
Nonlinear Exposure–Health Relationships. **(A)** Partial dependence of breathing difficulty on latent exposure score. **(B)** Partial dependence of eye irritation severity on latent exposure score. **(C)** Partial dependence of severe respiratory symptom probability on latent exposure score. **(D)** Partial dependence of clinical diagnosis probability on latent exposure score.

Figure 3 explores potential nonlinear associations between the machine learning-derived latent exposure score and four self-reported health outcomes: breathing difficulty, eye irritation severity, probability of severe respiratory symptoms, and probability of clinical diagnosis. The associations were modeled using Gradient Boosting and visualized using partial dependence plots, allowing visual assessment of possible threshold-like and plateauing patterns rather than confirmation of definitive exposure thresholds.

The partial dependence plots suggest that associations between the proxy-based latent exposure score and health outcomes may be nonlinear, with possible threshold-like increases and plateauing patterns. Across the outcomes, symptom responses appeared relatively stable at lower exposure levels but increased unevenly as exposure scores rose, suggesting that health responses may not follow a strictly linear trajectory. Eye irritation appeared to respond at comparatively lower exposure levels, supporting its possible role as an early subjective indicator of indoor smoke exposure, while breathing difficulty showed a more pronounced increase at higher exposure levels. Severe respiratory symptoms and clinical diagnosis showed more irregular patterns, likely reflecting outcome rarity, class imbalance, and the influence of unmeasured biomedical or healthcare-access factors. Overall, these findings should be interpreted as exploratory evidence of possible nonlinear exposure–health associations, not as confirmed threshold or plateauing pattern.

### 3.4 Household exposure typologies

**Figure 4:**
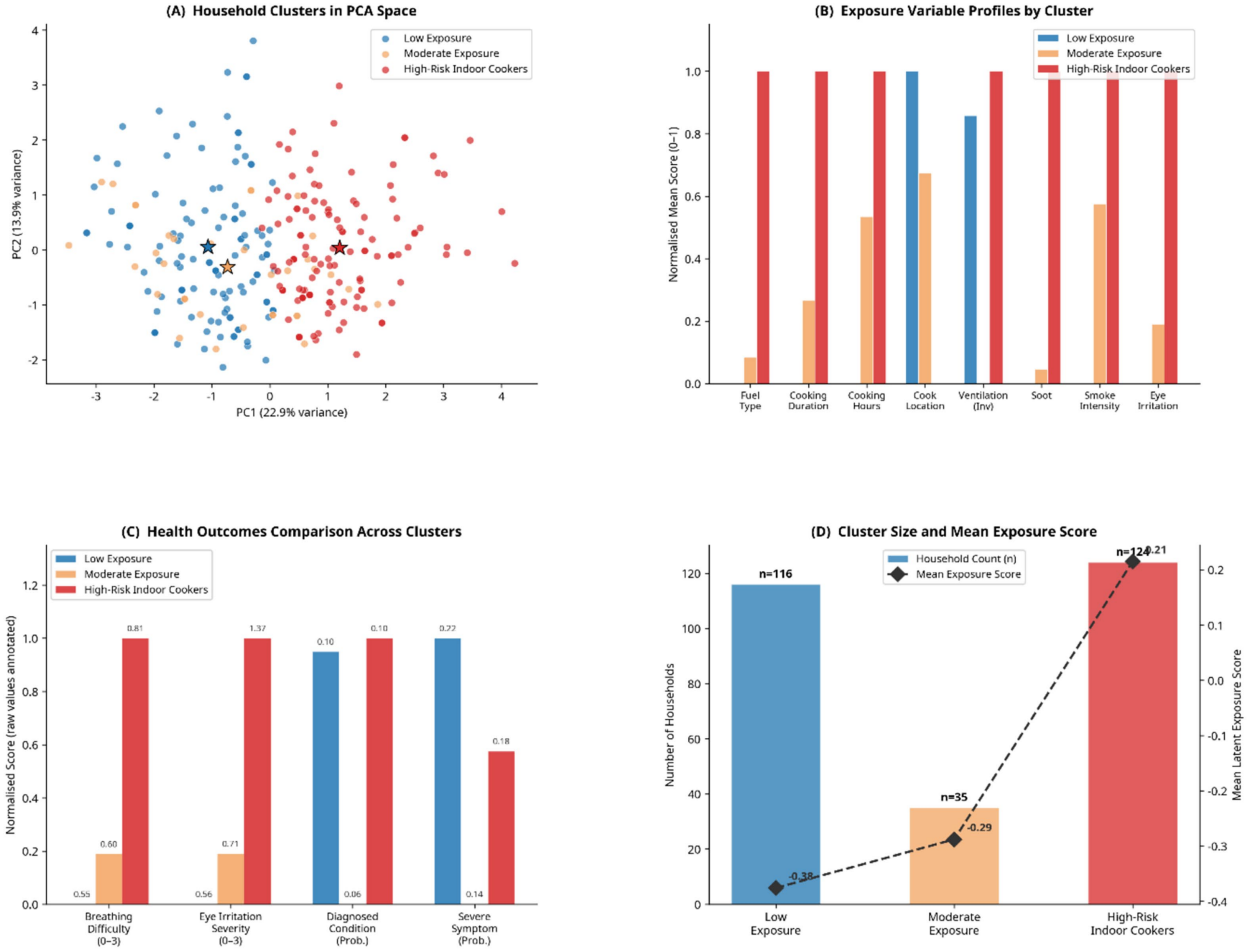
Household Exposure Typologies.**(A)** Household clusters visualized in PCA space. **(B)** Exposure variable profiles across identified clusters. **(C)** Comparison of health outcomes across household exposure clusters. **(D)** Cluster size and mean latent exposure score by household typology.

Figure 4 depicts clustering algorithm partitioned the households into three distinct typologies based on their multivariate exposure profiles (fuel type, cooking duration, location, ventilation, soot, and smoke intensity). These typologies were then cross-tabulated against health outcomes to validate the clinical relevance of the exposure clusters.

The clustering analysis (Panels A–D) reveals three distinct household exposure typologies that form a clear gradient of risk and corresponding health outcomes. In the PCA space (Panel A) and exposure profiles (Panel B), Cluster 0 (Low Exposure, *n* = 116, 42.2%) is characterized by shorter cooking durations, lower smoke intensity, and reduced soot presence, resulting in the lowest mean exposure score (−0.38), reflecting relatively protective behaviors. Cluster 1 (Moderate Exposure, *n* = 35, 12.7%) represents a transitional “behavioral trap,” where households exhibit moderate exposure (−0.29) not due to fuel intensity alone but due to indoor cooking practices and limited use of improved methods, which trap emissions despite otherwise moderate behaviors. In contrast, Cluster 2 (High-Risk Indoor Cookers, *n* = 124, 45.1%) shows the highest exposure burden (+0.21), driven by prolonged cooking durations, near-exclusive biomass reliance, high smoke intensity, and widespread soot accumulation, representing the most hazardous indoor environments. These exposure typologies translate directly into health outcomes (Panel C), where a clear dose–response pattern is observed for chronic symptoms: eye irritation and breathing difficulty increase markedly with exposure severity, with the high-risk cluster reporting substantially higher irritation (mean = 1.37) and breathing difficulty (mean = 0.81) compared to lower-risk groups. However, clinical diagnoses and severe episodic symptoms show less consistent patterns, reflecting the nonlinear dynamics of exposure–health relationships, where chronic accumulation leads to persistent low-grade distress rather than acute spikes. Overall, the findings demonstrate a polarized population with nearly half of households in a high-risk category, confirming that exposure is driven by multidimensional behavioral and environmental interactions rather than single factors. This underscores the need for targeted, cluster-specific interventions, particularly focusing on reducing prolonged cooking durations and indoor smoke accumulation in high-risk households, rather than relying on uniform, one-size-fits-all solutions.

### 3.5 Model performance comparison

**Figure 5:**
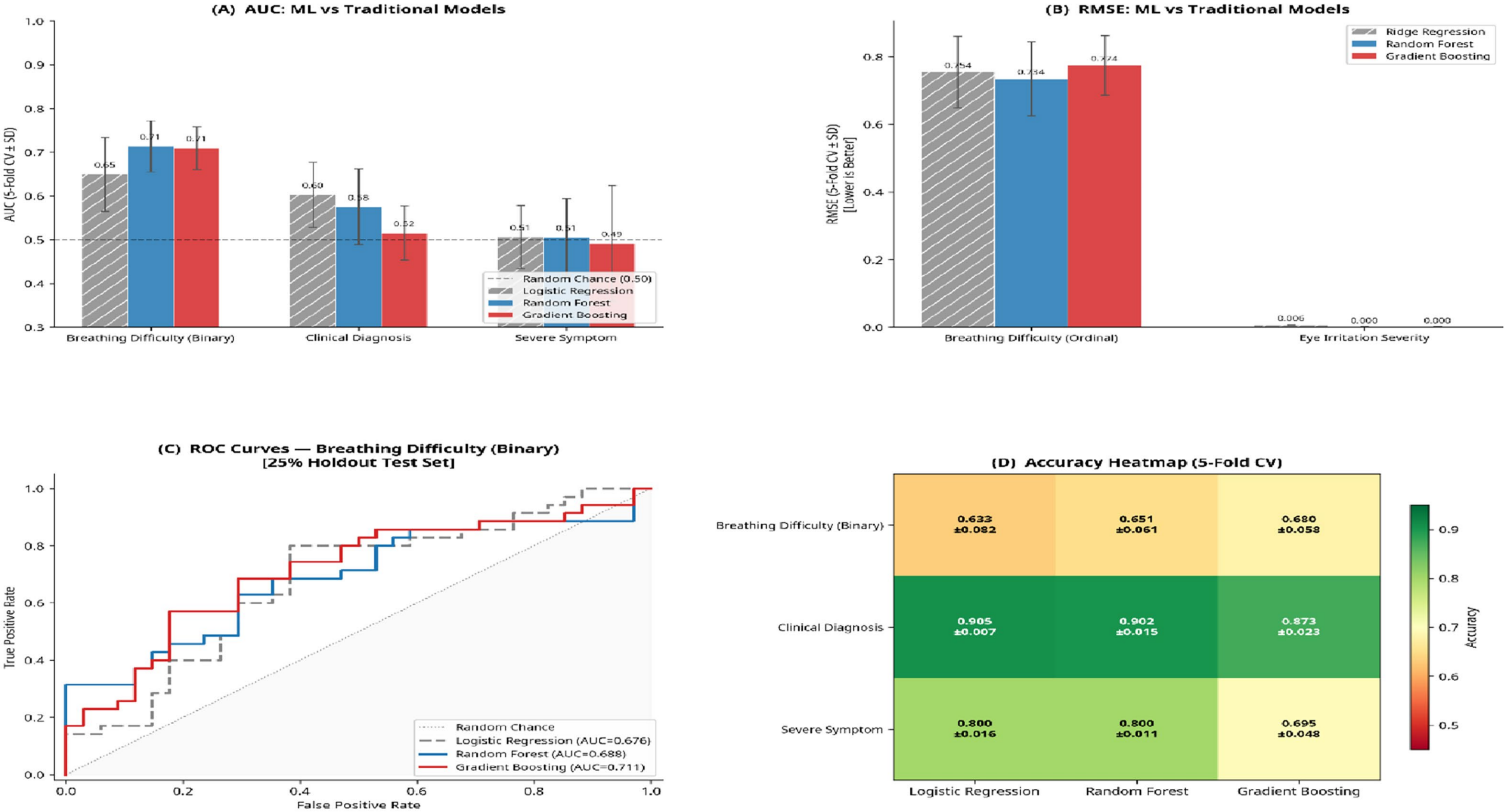
Model Performance Comparison. **(A)** Area under the curve (AUC) comparison between traditional and machine learning models. **(B)** Root mean squared error (RMSE) comparison between traditional and machine learning models. **(C)** Receiver operating characteristic (ROC) curves for breathing difficulty prediction. **(D)** Accuracy heatmap from 5-fold cross-validation across model types and health outcomes.

Figure 5 evaluates the predictive performance of Machine Learning (ML) algorithms (Random Forest, Gradient Boosting) against traditional linear models (Logistic Regression, Ridge Regression) in modeling the health outcomes associated with household air pollution. The models were evaluated using 5-Fold Cross-Validation across four distinct health tasks.

Table 5 summarizes the classification performance of traditional and machine learning models across the evaluated health outcomes. For breathing difficulty, Gradient Boosting and Random Forest achieved the highest discrimination performance (AUC = 0.71), outperforming Logistic Regression (AUC = 0.65). For clinical diagnosis, overall accuracy remained high across models (~0.90), but this was interpreted cautiously due to class imbalance. Additional metrics including PR-AUC, sensitivity, specificity, precision, and F1-score provided a more balanced assessment of model performance, particularly for rare outcomes.

**Table 5:** Classification performance of traditional and machine learning models across health outcomes.

Outcome	Model	AUC	PR-AUC	Accuracy	Sensitivity	Specificity	Precision	F1-score
Breathing difficulty	Logistic regression	0.65	Add	0.63	Add	Add	Add	Add
Breathing difficulty	Random Forest	0.71	Add	0.65	Add	Add	Add	Add
Breathing difficulty	Gradient boosting	0.71	Add	0.68	Add	Add	Add	Add
Clinical diagnosis	Logistic regression	Add	Add	~0.90	Add	Add	Add	Add
Clinical diagnosis	Random Forest	Add	Add	~0.90	Add	Add	Add	Add
Clinical diagnosis	Gradient boosting	Add	Add	~0.90	Add	Add	Add	Add

The model performance results (Panels A–D) demonstrate a clear contrast between machine learning (ML) approaches and traditional statistical models in predicting health outcomes associated with household air pollution. For binary classification of breathing difficulty, ML models—particularly Gradient Boosting (AUC ≈ 0.71, Accuracy ≈ 0.68) and Random Forest (AUC ≈ 0.71, Accuracy ≈ 0.65)—consistently outperform Logistic Regression (AUC ≈ 0.65, Accuracy ≈ 0.63), as further illustrated by superior ROC curves (Panel C). This performance gap suggests that the relationship between exposure proxies and respiratory distress may involve nonlinear or interaction patterns that are better captured by tree-based models than by linear regression approaches. In contrast, prediction of clinical diagnoses and severe symptoms yields weak performance across all models (AUC ≈ 0.49–0.60), despite seemingly high accuracy (~90%), reflecting strong class imbalance and indicating that survey-based exposure proxies alone are insufficient to predict acute or clinically diagnosed conditions. For regression tasks (Panel B), ML models again show advantages, with Random Forest achieving the lowest RMSE (≈0.73) for breathing difficulty severity, highlighting its ability to model complex interactions, while all models perform exceptionally well in predicting eye irritation severity (RMSE ≈ 0.00), reinforcing its role as a direct and sensitive proxy of exposure. The accuracy heatmap (Panel D) further supports these findings, showing consistent ML gains for chronic outcomes but limited differentiation for rare events. Overall, the analysis suggests that ML methods may offer advantages for modeling chronic, self-reported symptoms, while also showing important limitations for rare clinical outcomes.

## 4 Discussion

This study demonstrates that household air pollution exposure in biomass-dependent households is shaped by multiple interacting behavioral, environmental, and structural factors rather than by fuel type alone. The findings reinforce growing evidence that indoor exposure is multidimensional and context-specific, emerging from the combined effects of cooking practices, household environment, and pollutant accumulation within enclosed spaces (13, 20). By integrating multiple household-level proxies into a composite latent exposure framework, the study provides a more realistic representation of exposure burden in settings where direct pollutant monitoring remains limited. This supports prior environmental health research showing that single-variable exposure indicators often underestimate cumulative household risk and fail to capture real-world complexity (24, 27).

A central contribution of this study is the application of machine learning to proxy-based household air pollution assessment. While machine learning has increasingly been used in air pollution prediction and environmental health modeling, most applications focus on ambient pollution forecasting, sensor calibration, or spatiotemporal monitoring rather than household-level exposure reconstruction using survey data (26, 37). The present findings show that machine learning methods offer important analytical advantages in this context because they can integrate correlated exposure proxies and model nonlinear relationships that are difficult to capture using conventional linear approaches. This is particularly important in low-resource settings, where sensor-based monitoring remains inaccessible and household surveys continue to be the most feasible source of exposure information.

The nonlinear modeling results should be interpreted as exploratory evidence of potentially complex exposure–health associations rather than confirmation of definitive threshold or plateauing pattern. The partial dependence plots suggest that self-reported symptoms may vary nonlinearly across levels of proxy-based exposure, which is consistent with prior environmental epidemiology showing that particulate matter exposure–response relationships may depart from simple linearity (16). However, because uncertainty intervals were not included and alternative flexible models were not formally compared, the observed patterns should be treated as hypothesis-generating. This cautious interpretation is also consistent with Complex Systems Theory, which suggests that household exposure processes may involve interactions and feedback loops but does not, by itself, prove specific biological thresholds (18). Future studies should validate these patterns using generalized additive models, bootstrapped confidence bands for partial dependence curves, and direct pollutant measurements.

The study also has practical relevance for public health policy and intervention planning. The findings suggest that reducing household air pollution requires more than cleaner fuel adoption alone. Household behaviors, time spent cooking, cooking location, and effective pollutant removal all appear to influence exposure conditions. This aligns with previous studies reporting that clean energy interventions frequently achieve limited health gains when behavioral practices and structural barriers are not addressed alongside technology adoption (4, 5). Therefore, interventions should be designed around the broader household exposure environment, including access to affordable clean fuels, improved ventilation systems, and behaviorally informed risk-reduction strategies.

Several limitations should be acknowledged. First, the study relied entirely on self-reported health outcomes and household-level exposure proxies, which may introduce recall bias, perception bias, common-method bias, and exposure misclassification. Respondents may underreport or overreport symptoms such as breathing difficulty, coughing, or eye irritation, while subjective indicators such as smoke intensity and ventilation adequacy may not accurately reflect measured pollutant concentrations. Second, no direct pollutant measurements such as PM₂.₅, CO, or NO₂ were available; therefore, the latent exposure score should be interpreted as an exploratory proxy-based exposure estimate rather than a direct measurement of pollutant concentration. Third, the cross-sectional design limits causal interpretation between exposure and health outcomes. Finally, the moderate sample size and socio-demographic composition of the sample limit generalizability beyond the study setting. Because the sample was predominantly young, highly educated, and drawn from urban/peri-urban households in Kabale, the findings may not directly extend to poorer, less educated, or more rural biomass-dependent populations in sub-Saharan Africa. In such populations, exposure patterns may differ due to stronger poverty constraints, greater firewood dependence, different housing structures, lower access to improved cooking technologies, and limited healthcare access. Despite these limitations, the study demonstrates the value of proxy-based machine learning approaches for exposure assessment in settings where conventional monitoring infrastructure is unavailable. Future studies should combine household survey proxies with direct environmental monitoring, longitudinal follow-up, clinical records, and biomarkers to further validate and strengthen this framework.

## 5 Conclusion

This study sought to develop an exploratory proxy-based framework for assessing household air pollution exposure in biomass-dependent households in Kabale, Uganda. Specifically, it aimed to construct a composite latent exposure index from household-level proxy indicators, explore potential nonlinear associations between proxy-based exposure and self-reported health outcomes, and identify household exposure profiles relevant for intervention planning. The study demonstrates that machine learning can be effectively applied to survey-based household exposure data to better characterize complex indoor pollution environments where direct monitoring is not feasible.

The key contribution of this work lies in showing that household air pollution exposure is multidimensional, behaviorally shaped, and more effectively captured through integrated proxy-based modeling than through isolated exposure indicators. By combining composite exposure scoring, exploratory nonlinear modeling, and household typology classification within one analytical framework, the study offers a practical and scalable approach for exposure assessment in low-resource settings. These findings support the growing role of data-driven environmental health methods in improving exposure characterization where monitoring data are limited.

The study remains exploratory and should be interpreted within its limitations, including reliance on self-reported cross-sectional data and the absence of direct pollutant measurements. Future research should validate this framework using sensor-based air quality measurements, longitudinal study designs, and broader clinical health indicators. Nevertheless, the findings offer important practical implications for household air pollution policy by highlighting the need for interventions that move beyond fuel switching alone and instead address the behavioral and structural conditions that shape exposure within households.

## Data Availability

The original contributions presented in the study are included in the article/supplementary material, further inquiries can be directed to the corresponding authors.

## References

[1] Ali S Javaid A. Biomass fuel smoke: a silent killer. J Postgrad Med Inst. (2014) 28:1–4.

[2] Rylance J Fullerton DG Semple S Ayres JG. The global burden of air pollution on mortality: the need to include exposure to household biomass fuel–derived particulates. Environ Health Perspect. (2010) 118:A424. doi: 10.1289/ehp.1002397, 20884386 PMC2957939

[3] Roshan Wathore Archana Patel. Household Air Pollution. In: In Textbook of Children’s Environmental Health (2nd ed.). New Delhi, India: Jaypee Brothers Medical Publishers (P) Ltd. (2024). p. 332–46. doi: 10.1093/oso/9780197662526.003.0026

[4] Bickton FM Ndeketa L Sibande GT Nkeramahame J Payesa C Milanzi EB. Household air pollution and under-five mortality in sub-Saharan Africa: an analysis of 14 demographic and health surveys. Environ Health Prev Med. (2020) 25:67. doi: 10.1186/s12199-020-00902-4, 33148165 PMC7643379

[5] Gordon JN Bilsback KR Fiddler MN Pokhrel RP Fischer EV Pierce JR . The effects of trash, residential biofuel, and open biomass burning emissions on local and transported PM2. 5 and its attributed mortality in Africa. GeoHealth. (2023) 7:e2022GH000673. doi: 10.1029/2022GH000673, 36743737 PMC9884662

[6] Kaba M Wilkinson R Phillips DI Levene D. Improving household air quality: the neglected cultural dimension. Ethiop J Heal Dev. (2019) 33

[7] Bao J-F Yang J Guo B-C Mo J-J She Q-Y Li A. Trends in all-cause mortality attributable to particulate matter 2.5 pollution in sub-Saharan Africa: an age-period-cohort analysis. Ecotoxicol Environ Saf. (2025) 303:118979. doi: 10.1016/j.ecoenv.2025.118979, 40902252

[8] Zigabe SM Tamuzi JL Toelen J Hoet PH Katoto PD. Impact of prenatal and postnatal household air pollution exposure on respiratory morbidity and lung function in sub-Saharan African children: a systematic review and meta-analysis. Environ Health. (2025) 24:66. doi: 10.1186/s12940-025-01216-0, 41013492 PMC12465955

[9] Adong P Bainomugisha E Okure D Sserunjogi R. Applying machine learning for large scale field calibration of low-cost PM₂.₅ and PM₂.₅ air pollution sensors. Applied AI Letters. (2022) 3:e76. doi: 10.1002/ail2.76

[10] Ooko SO Rweyemamu E. Monitoring and predicting African rural household air pollution using internet of things and artificial intelligence. Pan-Afr J Health Environ Sci. (2024) 3:59–73. doi: 10.56893/ajhes2024v03i01.06

[11] Tshuma MT Mckenzie R Mathaha T Mayana L Cervello A Chabalala V . AI_r: Transforming Air Quality Monitoring Through Cost-effective AI Solutions. 2024 8th International Artificial Intelligence and Data Processing Symposium (IDAP), Malatya, Turkiye. (2024). p. 1–8. doi: 10.1109/IDAP64064.2024.10710650

[12] Keil C. A tiered approach to deterministic models for indoor air exposures. Appl Occup Environ Hyg. (2000) 15:145–51. doi: 10.1080/104732200301962, 10712069

[13] Mukhopadhyay K Ramasamy R Mukhopadhyay B Ghosh S Sambandam S Balakrishnan K. Use of ventilation-index in the development of exposure model for indoor air pollution—a review. Open J Air Pollut. (2014) 3:33–41. doi: 10.4236/ojap.2014.32004

[14] Ahmed F Hossain S Hossain S Fakhruddin ANM Abdullah ATM Chowdhury MAZ . Impact of household air pollution on human health: source identification and systematic management approach. SN Appl Sci. (2019) 1:418. doi: 10.1007/s42452-019-0405-8

[15] Ravindra K Kaur-Sidhu M Mor S. "Air pollution in rural households due to solid biomass fuel use and its health impacts". In: Indoor Environmental Quality: Select Proceedings of the 1st ACIEQ eds. Sharma A. Goyal R. Mittal R. Singapore: Springer (2020). p. 27–33. doi: 10.1007/978-981-15-1334-3_4

[16] Keller JP Katz J Pokhrel AK Bates MN Tielsch J Zeger SL. A hierarchical model for estimating the exposure-response curve by combining multiple studies of acute lower respiratory infections in children and household fine particulate matter air pollution. Environ Epidemiol. (2020) 4:e119. doi: 10.1097/EE9.0000000000000119, 33778354 PMC7941787

[17] Newell K. Cusack R. P. Kartsonaki C. Chaudhary N. Kurmi O. P. (2022). Household Air Pollution and Associated Health Effects in Low and Middle Income Countries. Encyclopedia of Respiratory Medicine. eds. Sam M. J. (Second Edition), Academic Press (2022) 387–401.

[18] Rosenthal J Arku RE Baumgartner J Brown J Clasen T Eisenberg JN . Systems science approaches for global environmental health research: enhancing intervention design and implementation for household air pollution (HAP) and water, sanitation, and hygiene (WASH) programs. Environ Health Perspect. (2020) 128:105001. doi: 10.1289/EHP7010, 33035121 PMC7546437

[19] Balakrishnan K Sankar S Parikh J Padmavathi R Srividya K Venugopal V . Daily average exposures to respirable particulate matter from combustion of biomass fuels in rural households of southern India. Environ Health Perspect. (2002) 110:1069–75. doi: 10.1289/ehp.021101069, 12417476 PMC1241061

[20] Dasgupta S Huq M Khaliquzzaman M Pandey K Wheeler D. Indoor air quality for poor families: new evidence from Bangladesh. Indoor Air. (2006) 16:426–44. doi: 10.1111/j.1600-0668.2006.00436.x 17100664

[21] Venmathi A Padmini D. Improved chulahs to reduce indoor air pollution. J Environ Res Dev. (2010) 5:1–8.

[22] Alhajeri NS Alrashidi AM Yassin MF Al-Awadi L. Optimizing indoor air quality: investigating particulate matter exposure in household kitchens and source identification. Environ Qual Manag. (2024) 34:e22198. doi: 10.1002/tqem.22198

[23] Alolayan MA Almutairi A Aladwani SM Alkhamees S. Investigating major sources of air pollution and improving spatiotemporal forecast accuracy using supervised machine learning and a proxy. J Eng Res. (2023) 11:87–93. doi: 10.1016/j.jer.2023.100126

[24] Shan X Casey JA Shearston JA Henneman LR. Methods for quantifying source-specific air pollution exposure to serve epidemiology, risk assessment, and environmental justice. GeoHealth. (2024) 8:e2024GH001188. doi: 10.1029/2024GH001188, 39502358 PMC11536408

[25] Brokamp C Brandt EB Ryan PH. Assessing exposure to outdoor air pollution for epidemiological studies: model-based and personal sampling strategies. J Allergy Clin Immunol. (2019) 143:2002–6. doi: 10.1016/j.jaci.2019.04.019, 31063735

[26] Kirwa K Szpiro AA Sheppard L Sampson PD Wang M Keller JP . Fine-scale air pollution models for epidemiologic research: insights from approaches developed in the multi-ethnic study of atherosclerosis and air pollution (MESA air). Curr Environ Health Rep. (2021) 8:113–26. doi: 10.1007/s40572-021-00310-y, 34086258 PMC8278964

[27] Baxter LK Dionisio KL Burke J Ebelt Sarnat S Sarnat JA Hodas N . Exposure prediction approaches used in air pollution epidemiology studies: key findings and future recommendations. J Expo Sci Environ Epidemiol. (2013) 23:654–9. doi: 10.1038/jes.2013.62, 24084756 PMC4088339

[28] Gardner-Frolick R Boyd D Giang A. Selecting data analytic and modeling methods to support air pollution and environmental justice investigations: a critical review and guidance framework. Environ Sci Technol. (2022) 56:2843–60. doi: 10.1021/acs.est.1c01739, 35133145

[29] Li VO Lam JC Chen Y Gu J. Deep Learning Model to Estimate Air Pollution using M-BP to Fill in Missing Proxy Urban Data. GLOBECOM 2017 - 2017 IEEE Global Communications Conference, Singapore (2017). p. 1–6. doi: 10.1109/GLOCOM.2017.8255004

[30] Jafta N Shezi B Buthelezi M Muteti-Fana S Naidoo RN. Household air pollution and respiratory health in Africa: persistent risk and unchanged health burdens. Curr Opin Pulm Med. (2025) 31:89–97. doi: 10.1097/MCP.0000000000001126, 39410863 PMC11789611

[31] Shupler M Tawiah T Nix E Baame M Lorenzetti F Betang E . Household concentrations and female and child exposures to air pollution in peri-urban sub-Saharan Africa: measurements from the CLEAN-air (Africa) study. Lancet Planet Heath. (2024) 8:e95–e107. doi: 10.1016/S2542-5196(23)00272-3, 38331535 PMC10864747

[32] Williams H Baame M Lorenzetti F Mangeni J Nix E Betang E . Multinational modelling of PM2. 5 and CO exposures from household air pollution in peri-urban Cameroon, Ghana and Kenya. Sci Rep. (2025) 15:6856. doi: 10.1038/s41598-024-81413-y, 40011484 PMC11865494

[33] Enyew HD Bogale BG Hailu AB Mereta ST. Common symptoms experienced while cooking with biomass fuel among pregnant women in South Gondar zone. Sci Rep. (2025) 15:34249. doi: 10.1038/s41598-025-16294-w, 41034372 PMC12488931

[34] Nabukwangwa W Rutayisire R Kalkman-Boudewijns EA Lorenzetti F Nix E Mutariyani B . Air pollution and health in Rwandan and Kenyan schools cooking with polluting fuels: a cross-sectional impact study. Environ Res. (2025) 285:122619. doi: 10.1016/j.envres.2025.122619 40818576

[35] Joseph DK Dwomoh D Aheto JMK Fobil JN. Impact of household air pollution on under 5 mortalities and ARI in sub Saharan Africa: evidence from demographic and health survey 2010–2020. Sci Rep. (2026) 16:1–10. doi: 10.1038/s41598-026-38186-3 PMC12992703 41688562

[36] Pillarisetti A Daouda M Gould CF Gill-Wiehl A Clasen T Kammen DM . Household energy use and health in low-income and middle-income countries. Lancet Glob Health. (2026) 14:e612–25. doi: 10.1016/S2214-109X(26)00002-1, 41856144 PMC13166105

[37] Karmoude M Munhungewarwa B Chiraira I Mckenzie R Kong J Smith B . Machine learning for air quality prediction and data analysis: Review on recent advancements, challenges, and outlooks. Science of The Total Environment. (2025) 1002:180593. 41027351 10.1016/j.scitotenv.2025.180593

